# Syntheses and characterization of two novel tetranuclear lead(II) clusters self-assembled by hydrogen bonded interactions

**DOI:** 10.1186/1752-153X-8-39

**Published:** 2014-06-23

**Authors:** Samson Jegan Jennifer, Packianathan Thomas Muthiah

**Affiliations:** 1 School of Chemistry, Tiruchirappalli 620024 Tamil Nadu, India

**Keywords:** Pb(II) clusters, 5-chloro thiophene 2-carboxylic acid, Pb(II) complex, Halogen involved hydrogen bonding

## Abstract

**Background:**

The usage of polynuclear metal clusters as secondary building units (SBU’s) in designing of metal organic frameworks (MOF’s) is a field of current interest. These metal clusters have attracted a great deal of attention not only due to their interesting structural topologies but also due to promising physical and chemical properties. In this regard various d,f block (transition and lanthanide) metal clusters have been widely investigated so far. Less attention is paid to construction of heavy p-block Pb(II) clusters.

**Results:**

Two mixed ligand Pb(II) clusters have been synthesized with bipy(2,2’-Bipyridine), phen(1,10-Phenanthroline), quin (8-Hydroxy quinolinate) and 5-tpc (5-chloro thiophene 2-carboxylate). They have been characterized by elemental analysis, IR, TGA and X-ray crystallography. X-ray diffraction analysis reveals that the complexes [Pb_4_(quin)_4_(bipy)_2_(5-tpc)_4_] (1) and [Pb_4_(quin) _4_(phen) _2_(5-tpc)_4_] **(2)** are tetranuclear. The complexes show a slight variation in unit cell parameters, due to the replacement of bipy and the phen ligands. Both complexes contain two types of Pb(II) ions which differ in the coordination geometry around the Pb(II) ion.

**Conclusions:**

In both complexes the four lead ions Pb1,Pb2, Pb1^i^ and Pb2^i^ lie on the same plane bridged by the 5-tpc anions. Pb1 and Pb2 of both complexes contain a 5-tpc and quin coordinated in a bidentate chelating bridging fashion. In addition the Pb2 and Pb2^i^ ions alone contain a bipy and phen in a bidentate chelating fashion in **(1)** and **(2)** respectively. An additional notable feature in both of these complexes are the bridging ability of the quin oxygen which forms a network of coordination bonds in between the four Pb(II) ions. In both complexes the individual units are self-assembled by C-H---Cl/C-H---S hydrogen bonding interactions to generate 2-D aggregates.

## Background

Although metal clusters show a lot of superiorities, the synthesis of unique metal clusters is a real challenge. Recent reports show that there are two strategies which can be employed in the design of metal clusters. They are usage of multidentate carboxylates and usage of mixed ligands [1-14]. In the mixed ligand approach a small multidentate ligand as well as a long bridging ligand is used, where the small multidentate ligand connects the metal ions to form the polynuclear cluster and the long bridging ligand can bridge these clusters.

The presence of an additional ligand may also lead to interesting structures with fascinating architectures. In our previous efforts to investigate the formation of Pb(II) complexes of quin and Pb(II) complexes of various N,N’-donating ligands we have already identified various intriguing structures with appealing supramolecular architectures [15,16]. We have also identified 5-tpc and quin as versatile multidentate ligands which can be used in the formation of metal complexes with interesting halogen bonding interactions [15-22]. As an extension of our previous work, in this study, we have investigated the complex formation of Pb(II) ions in the presence of mixed ligands (quin,phen/bipy) as well as 5-tpc and successfully obtained two novel complexes [Pb_4_(quin)_4_(bipy)_2_(5-tpc)_4_] (**1**) and [Pb_4_(quin)_4_(phen)_2_(5-tpc)_4_] (**2**) (bipy = 2,2’-Bipyridine, phen = 1,10-Phenanthroline, quin = 8-Hydroxy quinolinate and 5-tpc = 5-chloro thiophene 2-carboxylate) with similar cell parameters.

## Experimental section

### Materials and methods

Commercial starting materials were used without further purification. 2,2’bipyridine (Aldrich),5-Chloro thiophene 2- carboxylic acid (Hoechst Aktiengesellschaft), methanol (Qualigens, India), 8-hydroxyquinoline (Loba Chemie), Pb(CH_3_COO)_2_.3H_2_O (Reidel) were used. IR spectra of the complex in region 400–4000 cm^−1^ were recorded as pressed disks (1% by weight in KBr) on a Shimadzu FT IR spectrophotometer. Thermal stability studies were carried out on a STA 409 PL Luxx thermal analyzer at a heating rate of 10°C/min under nitrogen atmosphere. The fluorescent properties were studied in solid state on a HITACHI spectrofluorimeter in solid state at room temperature. Both the excitation slit and emission slit were 5 nm.

### Preparation of [Pb_4_(quin) _4_(bipy) _2_(5-tpc)_4_] (1)

A solution of Pb(CH_3_COO)_2_.3H_2_O (0.098 g) in 10 ml of (1:1) CH_3_OH/H_2_O mixture was stirred over a hot plate magnetic stirrer for half an hour and 5-Chloro thiophene 2-Carboxylic acid (0.0833 g) dissolved in 10 ml of CH_3_OH was added to it. A hot methanol solution of 8-hydroxyquinolin (0.0543 g) was added to it. The mixture was stirred for an additional of 2 hours. A yellow colored solution was formed. About (0.0442 g) of (2-2’-bipyridine) was dissolved in 10 ml of hot water and added to the reaction mixture; to this solution about (5 ml) of glacial acetic acid was added. The mixture was stirred for 3 hours. The dirty white precipitate was filtered off and the resulting pale yellow solution was kept for slow evaporation. After 3 days, pale yellow colored crystals suitable for X-ray diffraction were obtained. The crystals were filtered and washed with small portions of methanol and were dried in air (yield 75% based on Pb). IR selected bands (cm^−1^): 2924(m), 2654(m), 1564(s), 1527(s), 1492(s), 1460(s), 1427(s), 1367(s), 1319(s), 1279(s), 1103(s), 993(m), 671(m), 601(m), 493(m). Elemental analysis found: C 40.65%; H 2.61%; N 5.00%; S 5.62%; calculated: C 40.85%; H 2.71%; N 5.01%; S 5.74%.

### Preparation of [Pb_4_(quin) _4_(phen) _2_(5-tpc)_4_] (2)

The structure of complex **(1)** inspired us to design the preparation of complex **(2)** with same chelating mode using the 1,10-Phenanthroline ligand. The procedure of preparation of **(2)** is similar to **(1)**. Instead of 2-2’-bipyridine, 1,10-Phenanthroline was used (yield 62% based on Pb). IR selected bands (cm^−1^): 3045(m), 2924(m),1560(s), 1448(s), 1427(s), 1367(s), 1319(s), 1278(s), 1230(s), 1138(m), 1105(s), 1055(m), 993(m), 844(s), 821(s), 798(s), 765(s), 729(s), 669(s), 601(s),509(m), 491(s). Elemental analysis found: 42.07%; H 2.55%; N4.79%; S 5.58%; calculated: C 42.10%; H 2.65%; N4.91%; S 5.62%.

### Characterization of the complex IR spectra

On the basis of literature evidences assignment of selected characteristic IR bands (4000-400 cm^−1^) of the two Pb(II) complexes has been carried out. The spectra of the lead(II) complexes (**1**) and (**2**) were essentially similar and clearly show the carboxyl stretching vibrations of the 5-tpc. The asymmetric and symmetric stretches *ν*_as_(COO^−^) and *ν*_s_(COO^−^) were observed at 1564 and 1367 cm^−1^, 1560 and 1367 cm^−1^ for the complexes (**1**) and (**2**) respectively. The Δ = *ν*_as_(COO^−^) − ν_s_(COO^−^) = 197 cm^−1^ and 193 cm^−1^ for the complexes (**1**) and (**2**) respectively. These observed values are only a slightly higher than those expected for ionic carboxylate group in acetates (170 cm^−1^) [23].

### Crystal structure determination

Intensity data sets were collected at room temperature, on a BRUKER SMART APEXII CCD [24] area-detector diffractometer equipped with graphite monochromated Mo Kα radiation (λ = 0.71073 Å). The data were reduced by using the program SAINT [24] and empirical absorption corrections were done by using the SADABS [24]. The structures were solved by direct methods using SHELXS-97 [25] and refined anisotropically by full-matrix least-squares method using SHELXL-97 [25] within the WINGX suite of software, based on F^2^ with all reflections. All carbon hydrogens were positioned geometrically and refined by a riding model with U_iso_ 1.2 times that of attached atoms. All non H atoms were refined anisotropically. The molecular structures were drawn using the ORTEP-III [26] and POV-ray [27]. Crystal data and the selected parameters are summarized in (Tables 1 and 2) respectively. The crystals remained stable throughout the data collection. The CIF files of complexes 1 and 2 ate provided as Additional files 1 and 2 respectively.

**Table 1:** Crystal data and refinement parameters

	**Complex 1**	**Complex 2**
Empirical formula	C76 H48 Cl4 N8 O12 Pb4 S4	C80 H48 Cl4 N8 O12 Pb4 S4
Formula weight	2364.11	2412.15
Temp, K	296	296
λ (Å)	0.71073	0.71073
Crystal system	Triclinic	Triclinic
Space group	P-1	P-1
a (Å)	11.6469(4)	11.5185(2)
b (Å)	12.2824(4)	12.2250(2)
c (Å)	14.1122(4)	14.7743(2)
α(°)	82.427(2)	81.913(1)
β (°)	72.489(2)	72.349(1)
γ (°)	84.915(2)	84.455(1)
V (Å3)	1905.87(11)	1959.55(5)
Z	1	1
ρ calcd (g/cm3)	2.060	2.044
μ (mm-1)	9.124	8.876
F (000)	1116	1140
Crystal size (mm)	0.09 × 0.11 × 0.12	0.08 × 0.09 × 0.09
No of reflections collected	12353	31600
Number restraints	0	0
Goodness-of-fit on F2	1.01	1.18
Final R1 index [I > 2σ(I)]	0.0381	0.0232
wR2 (all data)	0.1054	0.0618
Largest difference in peak and hole (e Å-3)	−1.64, 2.92	−0.65, 2.61
CCDC number	954993	954992

R1 = ∑(||Fo|-|Fc||)/∑| Fo |; wR2 = [∑w(|Fo|-|Fc|2)2]/∑w(|Fo|2)]1/2.

**Table 2:** Selected bond lengths (Å) and bond angles (°) for complexes (1) and (2)

**Selected bonds**	**Value (Å)**	**Selected angles**	**(°)**	**Selected bonds**	**Value (Å)**	**Selected angles**	**(°)**
**Complex 1**				**Complex 2**			
Pb1-O1	2.498(4)	O1 -Pb1-O2	47.27(15)	Pb1-O1	2.341(3)	O1-Pb1-O2	71.10(12)
Pb1-O2	2.962(6)	O1 -Pb1-O3	71.84(15)	Pb1-O2	2.605(4)	O1-Pb1-O5	73.83(13)
Pb1-O3	2.342(4)	O1 -Pb1-O4	73.97(14)	Pb1-O5	2.497(4)	O1-Pb1-O6	117.20(11)
Pb1-O4	2.615(4)	O1 -Pb1-N1	76.85(17)	Pb1-O6	2.979(5)	O1-Pb1-N1	68.30(13)
Pb1-N1	2.483(5)	O1 -Pb1-O3^i^	140.79(14)	Pb1-N1	2.482(4)	O1-Pb1-O1^i^	71.84(10)
Pb1-O3^i^	2.607(4)	O1 -Pb1-O6^i^	141.42(15)	Pb1-O1^i^	2.637(3)	O1-Pb1-O4^i^	116.78(13)
Pb1-O6_a	2.812(5)	O2-Pb1-O3	115.33(15)	Pb1-O4^i^	2.832(4)	O2-Pb1-O5	73.87(11)
Pb2-O1	3.040(5)	O2-Pb1-O4	104.81(14)	Pb2-O1	2.848(3)	O2-Pb1-O6	103.69(12)
Pb2-O3	2.857(4)	O2-Pb1-N1	77.78(16)	Pb2-O2	2.349(3)	O2-Pb1-N1	134.56(12)
Pb2-O4	2.338(4)	O2-Pb1-O3^i^	171.93(13)	Pb2-O3	2.687(5)	O1 ^i^ -Pb1-O2	81.26(10)
Pb2-O5	2.770(6)	O2-Pb1-O6^i^	102.58(15)	Pb2-O4	2.868(5)	O2-Pb1-O4^i^	145.95(10)
Pb2-O6	2.756(6)	O3-Pb1-O4	71.00(14)	Pb2-N2	2.448(4)	O5-Pb1-O6	46.74(13)
Pb2-N2	2.447(5)	O3-Pb1-N1	68.09(15)	Pb2-N3	2.782(4)	O5-Pb1-N1	76.03(14)
Pb2-N3	2.788(6)	O3-Pb1-O3^i^	72.19(13)	Pb2-N4	2.720(5)	O1 ^i^ -Pb1-O5	142.56(12)
Pb2-N4	2.747(6)	O3-Pb1-O6^i^	116.12(16)			O4 ^i^ -Pb1-O5	139.70(12)
		O4-Pb1-N1	135.28(15)			O6-Pb1-N1	78.19(14)
		O3 ^i^ -Pb1-O4	80.06(13)			O1 ^i^ -Pb1-O6	170.60(11)
		O4-Pb1-O6^i^	144.46(14)			O4 ^i^ -Pb1-O6	101.05(12)
		O3 ^i^ -Pb1-N1	103.45(14)			O1 ^i^ -Pb1-N1	104.19(13)
		O6 ^i^ -Pb1-N1	72.67(16)			O4 ^i^-Pb1-N1	73.38(13)
		O3 ^i^ -Pb1-O6^i^	70.52(14)			O1 ^i^ -Pb1-O4^i^	71.37(11)
		O1-Pb2-O3	57.53(11)			O1-Pb2-O2	66.61(11)
		O1-Pb2-O4	68.38(12)			O1-Pb2-O3	105.13(12)
		O1-Pb2-O5	151.53(15)			O1-Pb2-O4	67.93(11)
		O1-Pb2-O6	125.36(14)			O1-Pb2-N2	119.10(12)
		O1-Pb2-N2	131.30(14)			O1-Pb2-N3	158.84(12)
		O1-Pb2-N3	110.22(15)			O1-Pb2-N4	125.85(11)
		O1-Pb2-N4	69.02(14)			O2-Pb2-O3	131.56(13)
		O3-Pb2-O4	66.66(13)			Pb1-O1-Pb2	99.06(12)
		O3-Pb2-O5	106.96(13)			Pb1-O1-Pb1^i^	108.16(11)
		O3-Pb2-O6	67.83(13)			O2-C11-C10	118.8(4)
		O3-Pb2-N2	121.51(13)			O3-C31-O4	124.0(7)
		O3-Pb2-N3	156.42(15)			O5-C36-O6	124.6(5)
		O3-Pb2-N4	123.30(13)			Pb1-O2-Pb2	106.07(13)
		O4-Pb2-O5	131.07(15)			Pb1 ^i^-O4-Pb2	104.09(13)
		O4-Pb2-O6	91.33(16)				
		O4-Pb2-N2	68.81(14)				
		O4-Pb2-N3	130.95(17)				
		O4-Pb2-N4	77.90(16)				
		O5-Pb2-O6	46.86(15)				
		O5-Pb2-N2	76.77(16)				
		O5-Pb2-N3	74.20(17)				
		O5-Pb2-N4	129.58(15)				
		O6-Pb2-N2	77.43(16)				
		O6-Pb2-N3	120.39(17)				
		O6-Pb2-N4	157.57(15)				
		N2-Pb2-N3	81.96(16)				
		N2-Pb2-N4	80.33(15)				
		N3-Pb2-N4	58.44(17)				
		Pb1-O1-Pb2	90.98(14)				
		Pb1-O3-Pb2	99.01(13)				
		Pb1-O3-Pb1^i^	107.81(15)				
		Pb1 ^i^ -O3-Pb2	108.59(13)				
		Pb1 ^i^ -O3-C13	113.5(3)				
		Pb1 ^i^ -O6-Pb2	105.74(17)				
		O1-C1-O2	125.4(6)				
		O5-C34-O6	124.8(7)				

In Complex 1[symmetry code i = 1-x,1-y,1-z, in complex 2 [symmetry code i = 1-x,1-y,-z].

## Results and discussion

### Geometry around lead

**Figure 1:**
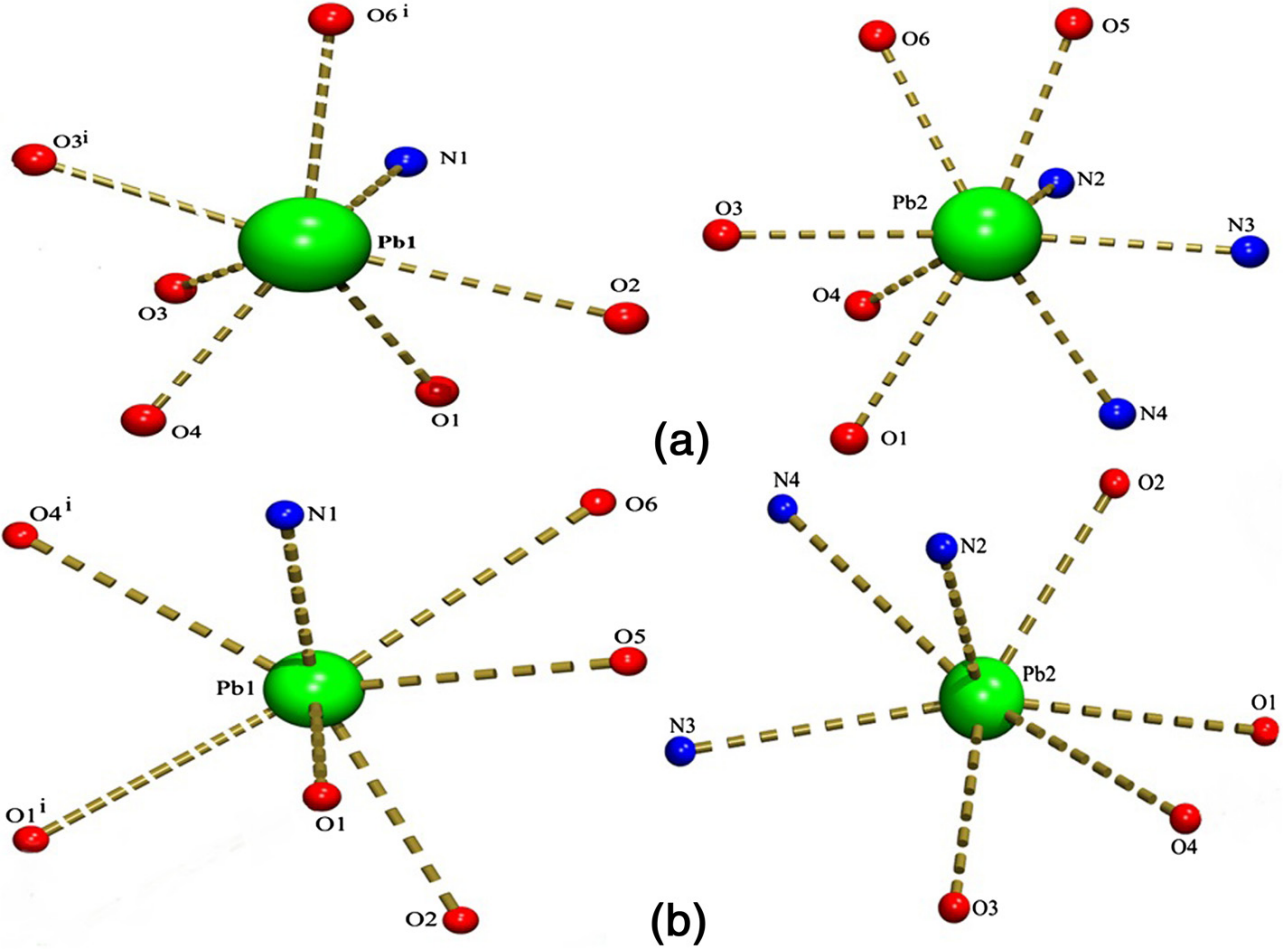
**Coordination environment around the two Pb(II) ions. (a, b)** Coordination environment in complexes (1,2) respectively.

It has been well studied that the lone pair of electrons has a great influence on the structure of the Pb(II) complexes [28-31]. In the coordination chemistry of the Pb(II) ion, the terms holo and hemi directed are used to describe the geometries around the central Pb atom [29]. Pb(II) complexes in which the bonds to ligand atoms are placed throughout the surface of the encompassing globe are said to be holo directed, while hemidirected refers to those cases in which the bonds to ligand atoms are directed throughout only part of an encompassing globe [32]. The hemidirected geometry is the most preferred for intermediate coordination numbers between 6–8 [31]. The coordination geometry of both the complexes (**1,2**) as well as the Pb-O and the Pb-N bond directions show a gap around the Pb(II) ion which is also well depicted from (Figure 1). In both the complexes, the O-Pb-O angle suggests that there is a big gap in the coordination sphere due to lone pair-bond pair repulsion (Table 2). This gap is occupied possibly by a stereoactive lone pair of electrons on the lead(II) ion. The coordination around the Pb(II) ions in both (**1, 2**) are hemidirected indicating that the stereochemical lone-pair electrons of them are active. The presence of a lone pair of the Pb(II) ions in both Pb1 and Pb2 in the direction opposite to that of the quinolate bridging is apparently the reason that the bridging ligand can come closer to the next Pb(II) ion.

### Crystal structure description of [Pb_4_(quin) _4_(bipy) _2_(5-tpc)_4_] (1) and [Pb_4_(quin) _4_(phen) _2_(5-tpc)_4_] (2)

**Figure 2:**
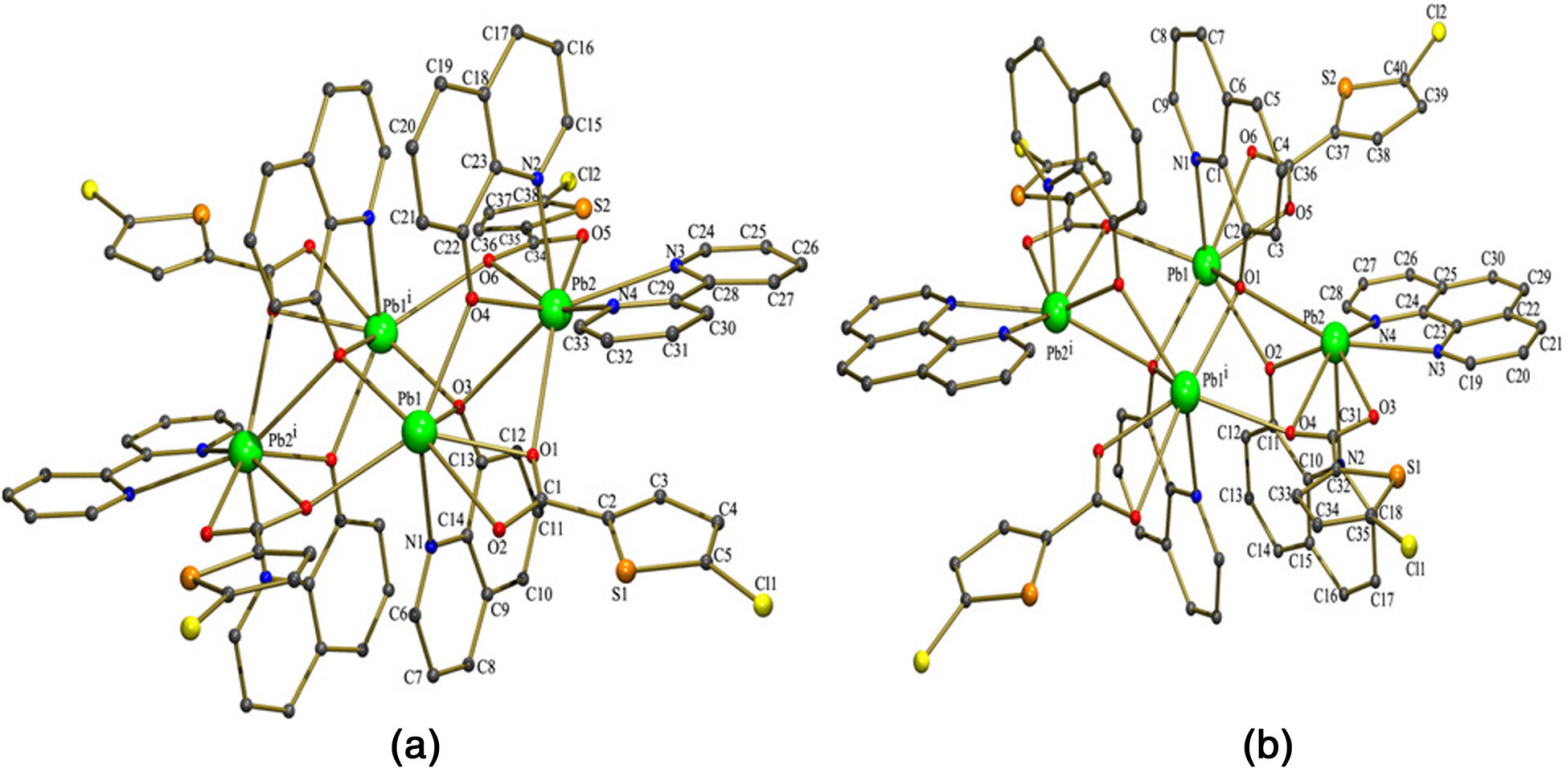
**Molecular structures of (1) and (2) displaying the crystallographic numbering scheme. (a, b)** Displacement ellipsoids are drawn at the 50% probability level. The hydrogen atoms of (1) are omitted for clarity and hydrogens of (2) are included as spheres of arbitrary radii.

The two crystals **(1)** and **(2)** are nearly isostructural and have similar unit cell parameters due to the similarity of the chelating ligands used (bipy, phen). In both the complexes the tetranuclear units has an inversion centre, coinciding with the crystallographic inversion centre. Pb1 is seven coordinated in both **(1)** and (**2**) but the Pb2 is eight coordinated in **(1)** and seven coordinated in **(2)** (Figure 1). Thus each crystal has two type unique Pb centres (Pb1 and Pb2) while the other two are generated by inversion (Figure 2). Both the complexes **(1)** and **(2)** are made up of isolated terameric units with [Pb_4_(μ-quin)_4_] as building blocks. The N and O atoms of four quin anions bridge four Pb(II) ions. The Pb…Pb distances in between the Pb ions in the tetramers are [Pb1-Pb2 = 3.9675(3) Å in 1, 3.9609(3) Å in **(2)**; Pb2-Pb1^i^ = 4.4393(3) Å in **(1)**, in 4.4943(3) Å **(2)**], which are in the similar range of previously reported Pb–Pb distances [29,30]. It is interesting that there is a weak Pb…Pb interaction between Pb1 and Pb2 in both **(1)** and **(2)**. The extent of direct Pb…Pb interactions has been rarely reported and there are a few reports of these interactions in between adjacent Pb atoms with distance range of 3.44-4.09 Å in the clusters [33-36]. The short Pb…Pb distances in both the complexes which are smaller than the sum of *van der Waals* radii of two Pb(II) atoms, suggest a possibly weak metallophilic Pb…Pb interaction [35,36]. In both the complexes the quin ligands acts as both bidentate and bridging ligand in a μ-1,4 mode where the oxygen of the of quin coordinates to the Pb(II) ion and also bridges the adjacent Pb(II) ions. There are four 5-tpc anions which act as one of the prime reason in deciding the coordination geometry around the Pb(II) ion. In **(1)** the 5-tpc acts only as a bidentate chelating group. But in **(2)** two different coordination modes like bidentate chelating and bidentate chelating bridging modes are observed.

**Figure 3:**
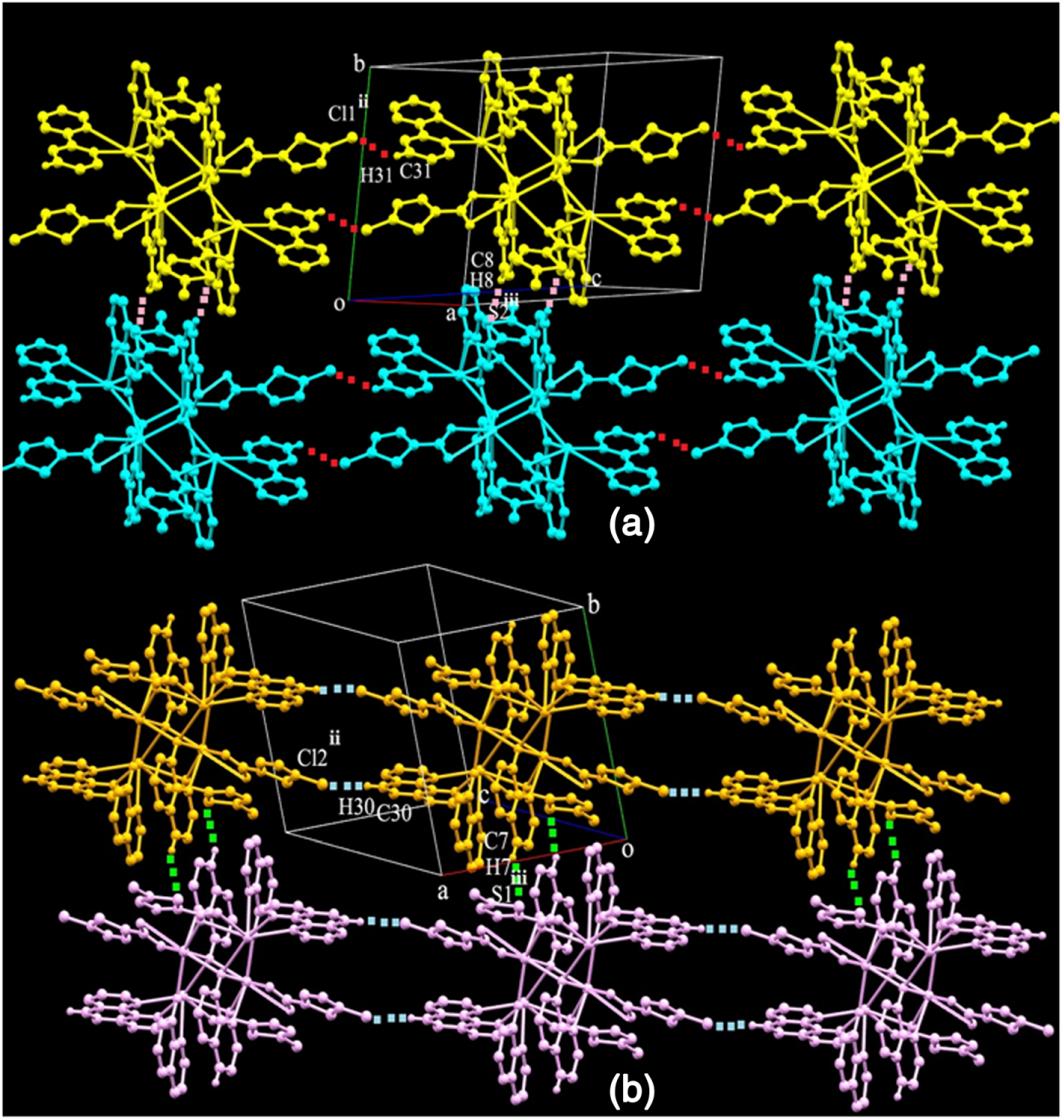
**C-H…Cl and C-H…S interactions in (1) and (2). (a,b)** Chain made up of C-H…Cl interactions [red –(1), blue-(2)] and C-H…S interactions [pink –(1), green-(2)]. The hydrogen atoms which are not involved in hydrogen bonding are omitted for clarity.

The mode of coordination of the 8-hydroxyquinolate ligand in the previously reported Pb(II) complexes, namely [Pb_2_(8-Quin)_2_(NO_3_)_2_(MeOH)] [31] and [Pb4(8-Quin)4(Tp)2(DMF)2]_n_[37] (where MeOH = Methanol, DMF = Dimethyl Formamide)is similar to the one found in complexes **(1)** and **(2)** (Figure 2). These isolated tetranuclear units are linked to a chain by a pair of C-H…Cl interactions in between of a tetranuclear unit and of the other (Figure 3). These interactions are found in between C31-H31∙∙∙Cl(1) [symmetry code: −x,1-y,-z] in **(1)** and C30-H30∙∙∙Cl2 [symmetry code: 2-x,1-y,1-z] in **(2)**. Further each of these individual chains is linked to each other by weak C-H…S interactions in between the S of the thiophene ring of one chain and H of quin ring of another chain, which leads to formation a 2D layer (Figure 3).

These 2D layers are further stacked in to 3D supramolecular architectures by π –π stacking interactions. In **(1)** these interactions are found in between five membered thiophene rings of one layer and bipyridine rings of the next layers (Cg1…Cg5[symmetry code: 1-X,1-Y,-Z] and Cg2…Cg6 [symmetry code: 1 + X,Y,Z] where Cg1 = S1,C2-C5;Cg2 = S2,C35-C38; Cg5 = N3,C24-C28; Cg6 = N4,C29-C33). In **(2)** the same kind of interactions are found in between thiophene rings of one layer and phen rings of adjacent layer (Cg1…Cg7[symmetry code: −1 + X,Y,Z] and Cg2…Cg6 [symmetry code: 1-X,1-Y,1-Z] where Cg1 = S1, C32-C35; Cg2 = S2,C37,C40,C39,C38; Cg7 = N4,C24-C28; Cg6 = N3,C19-C23). In addition to the above said interactions there are some π-π stacking interactions in between phen of nearby layers (Cg9…Cg8 [symmetry code: 1-X,-Y,-Z] and Cg9…Cg4 [symmetry code: 1-X,-Y,-Z] where C9 = C10-C15; Cg8 = C1-C6, Cg4 = N1, C1, C6-C9).

### Thermal stability of (1,2)

**Figure 4:**
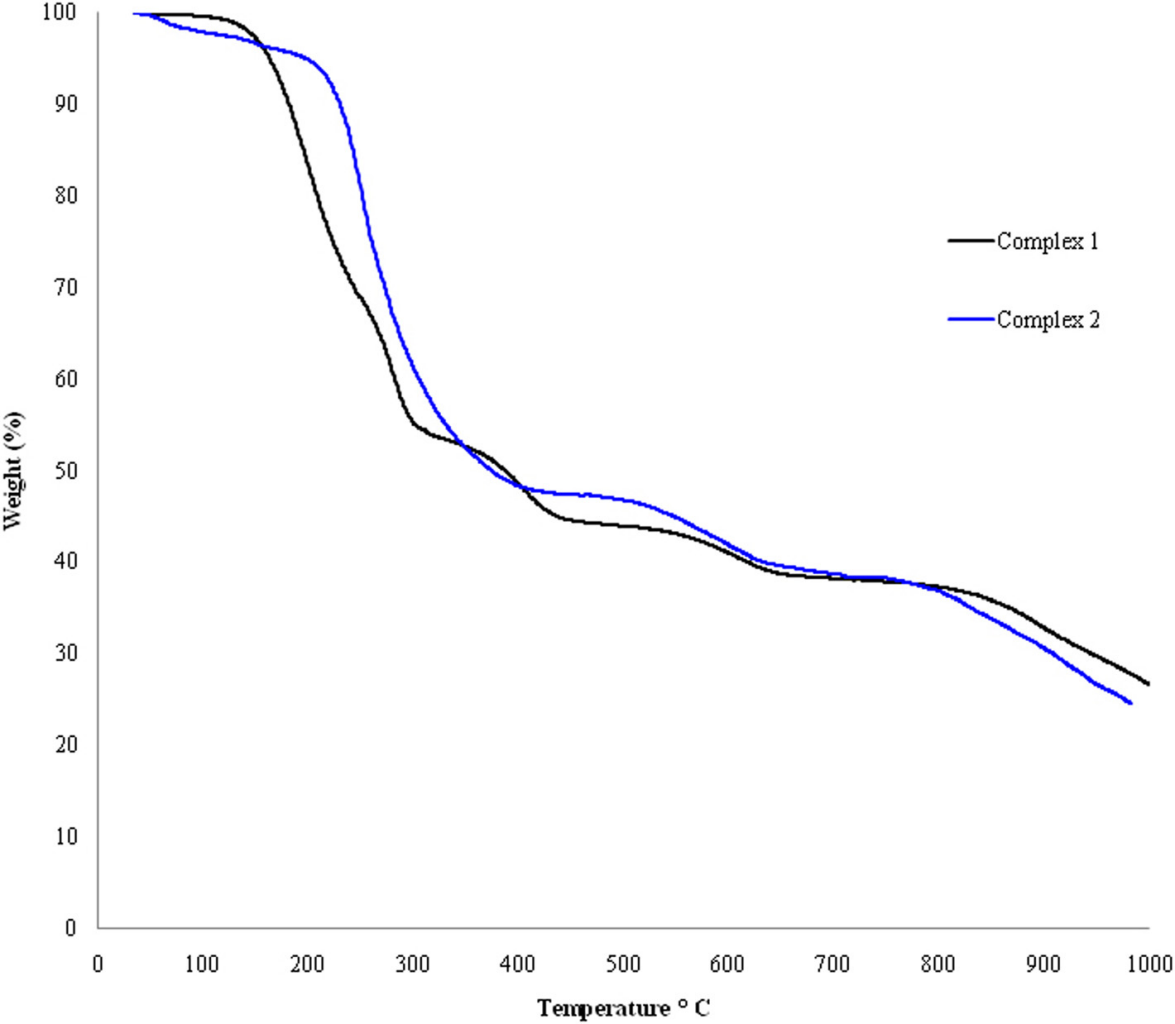
Thermograms of complexes (1,2) showing TGA curves at the heating rate of 10°C/min.

Thermo gravimetric analysis (TGA) experiments of the complexes **(1)** and **(2)** were conducted under a static atmosphere of nitrogen at temperatures ranging from RT (room temperature) to 1000°C in order to determine the thermal stabilities. Due to similarities of complexes **(1)** and **(2)** they show similar decomposition patterns (Figure 4). Both the complexes started to melt well above 170°C showing a very small thermal effect. Complexes **(1), (2)** showed three steps of thermal decomposition at (160-316°C, 170-353°C) which probably due to four 8quin ligands. The decomposition of the bipy and phen ligands took place at (320-608°C, 370-605°C) in **(1), (2)** respectively. Complexes **(1), (2)** exhibit their third weight loss at temperature ranges of (610-988°C and 610-989°C). This is due to loss of four 5-tpc molecules in both the complexes.

### Luminescent properties

**Figure 5:**
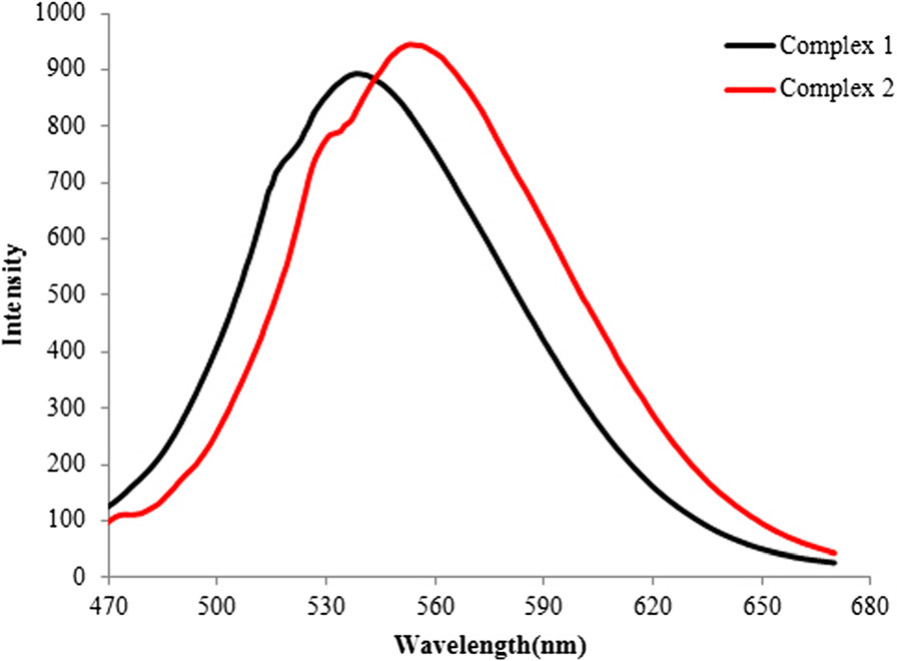
**Solid-state emission spectra of the complexes at room temperature (black: 1,λ**_**ex **_**= 459 nm; red: 2, λ**_**ex **_**= 460 nm).**

Luminescent properties of complexes **1** and **2** have been investigated in solid state at room temperature. It can be observed from the emission spectrum that complexes **1** and **2** exhibits intense and broad emission band with an emission maximum at ca. 543 and 552 nm upon excitation at 459, 460 nm (Figure 5). It is also observed that the emission spectra of **1** and **2** are similar. This emission band could be assigned to the emission of ligand-to-metal charge transfer (LMCT). This observation indicates that complexes **1** and **2** may be used as potential candidates for a new class of photoactive materials.

## Conclusion

Two novel tetranuclear Pb(II) complexes in concert with 5-tpc and corresponding bidentate chelating N.N’ ligands have been synthesized and characterized. Although both the complexes seem to be similar based on their tetranuclear Pb(II) ions, on considering their coordination geometry around their Pb(II) they are completely different. These complexes are also perfect examples to elucidate the bridging ability of the quin ligand. In both the crystal structures the C-H…Cl hydrogen bonding interactions play a major role in building up of the supramolecular architectures. However this work not only shows the influence of replacement of different ligands on the structure but also its consequence on the crystal structure and geometry of the central metal ion, which also provides valuable instruction in design of coordination complexes with desired supramolecular architectures.

## Competing interests

The authors declare that they have no competing interests.

## Authors’ contributions

This work was prepared in the research group of PTM. He proposed the work and drafted the manuscript. SJJ participated in the design and presiding the experiments, collected the X-ray data and drafted the manuscript. Both authors read and approved the final manuscript.

## Supplementary Material

Additional file 1 **CCDC 954993; CCDC 954992 contain the supplementary crystallographic data for complexes (1) and (2) and respectively can be obtained free of charge via **http://www.ccdc.cam.ac.uk/conts/retrieving.html**, or from the Cambridge Crystallographic Data Center, 12 Union Road, Cambridge CB2 IEZ, UK; fax:(+44)1223-336-033; or e-mail: deposit@ccdc.cam.ac.uk.** Click here for file

Additional file 2 **CCDC 954993; CCDC 954992 contain the supplementary crystallographic data for complexes (1) and (2) and respectively can be obtained free of charge via **http://www.ccdc.cam.ac.uk/conts/retrieving.html**, or from the Cambridge Crystallographic Data Center, 12 Union Road, Cambridge CB2 IEZ, UK; fax:(+44)1223-336-033; or e-mail: deposit@ccdc.cam.ac.uk.** Click here for file
